# Hip structural analysis: a comparison of DXA with CT in postmenopausal Japanese women

**DOI:** 10.1186/2193-1801-2-331

**Published:** 2013-07-20

**Authors:** Kazuhiro Ohnaru, Teruki Sone, Kensuke Tanaka, Kazumi Akagi, Yong-In Ju, Hak-Jin Choi, Tatsushi Tomomitsu, Masao Fukunaga

**Affiliations:** Department of Nuclear Medicine, Kawasaki Medical School, 577 Matsushima, Kurashiki, Okayama, 701-0192 Japan; Department of Health and Sports Sciences, Kawasaki University of Medical Welfare, 288 Matsushima, Kurashiki, Okayama, 701-0193 Japan; Department of Radiological Technology, Kawasaki College of Allied Health Professions, 316 Matsushima, Kurashiki, Okayama, 701-0194 Japan; Kawasaki Medical School, 577 Matsushima, Kurashiki, Okayama, 701-0192 Japan

**Keywords:** Hip structural analysis, CT-based finite-element method, Bone strength, QCT

## Abstract

Geometry of the proximal femur is one determinant of fracture risk, and can be analyzed by a simple method using dual-energy X-ray absorptiometry (DXA). The aim of the present study was to investigate the accuracy of hip structural analysis (HSA) using clinical data in postmenopausal Japanese women. A total of 184 postmenopausal women aged 51–88 years (mean, 70.5 ± 8.7 years) who underwent artificial joint replacement surgery for osteoarthrosis of the hip or knee joint were included. Computed tomography (CT) data from preoperative assessment were utilized for analysis of proximal femoral geometry (CT-HSA) using QCTPro Software (Mindways Software Inc., Austin, TX) and compared with HSA results based on DXA (DXA-HSA). The results of femoral geometry were further compared with a CT-based finite-element method (CT/FEM). There was moderate to high correlation between DXA-HSA and CT-HSA (r=0.60-0.90, p<0.001), except for the buckling ratio in the intertrochanteric region. Moreover, the correlation of HSA with CT/FEM was similar between DXA-HSA and CT-HSA. The present results suggest that the geometry of proximal femoral cross sections can be reasonably well characterized using DXA.

## Introduction

Japan is facing the major challenge of meeting the burgeoning health care needs of a rapidly aging population. Osteoporotic fracture is one of the common causes of elderly persons becoming bed-ridden in Japan, and is an issue that needs to be urgently addressed. Among common sites of osteoporotic fractures, the proximal femur can be one the most devastating. In the first year following hip fracture the mortality rate rises to 20-24% (Cooper et al. 1993; Leibson et al. 2002), and survivors of hip fracture often live with chronic pain, disability, and increasing dependence on caretakers (Keene et al. 1993). In Japan, approximately 150,000 hip fractures occur per year, and this number has continued to rise over the past 20 years (Orimo et al. 2009).

Assessment of bone strength is important in the diagnosis of osteoporosis. Although bone mineral density (BMD) correlates with bone strength and bone densitometry, especially dual-energy X-ray absorptiometry (DXA), is widely used to assess BMD in clinical practice (Cummings et al. 2002), it is well known that BMD itself is inadequate for accurate estimation of bone strength (Cody et al. 1999; Esses et al. 1989; Beck et al. 1990). DXA has limitations owing to the complex three-dimensional (3D) geometry of the hip, low spatial resolution, and the two-dimensional (2D) nature of this imaging approach. To overcome these limitations, Beck et al. have worked to derive more complex biomechanical indices based on hip structural analysis (HSA) (Beck et al. 1990). DXA-based HSA (DXA-HSA) is a simple and easy method that can be used to assess proximal femoral geometry. Although various investigations have been made on the clinical relevance of DXA-HSA, consensus has not been obtained because of the variations between races, evaluation methods, et al. Furthermore, limitations imposed by the 2-dimensional nature of DXA have not been fully addressed (Bouxsein and Karasik 2006). From the currently available data, the HSA structural parameters by DXA are highly correlated with areal BMD and while predictive of fracture risk, they have not shown much improvement in the fracture prediction compared to areal BMD. Nevertheless, HSA with DXA has provided unique insights into the mechanisms of both the pathophysiology of osteoporotic fracture and the therapeutic efficacy of bone-active agents.

The major limitations of HSA with DXA primarily reflect limitations imposed by the 2-dimensional nature of DXA. As an alternative to DXA, quantitative computed tomography (QCT) offers complete 3D information, high in-plane spatial resolution, bone geometry, and separate assessment of the cortical and trabecular bones of the femur (Bauer et al. 2006; Duchemin et al. 2008). Recently, CT technology has dramatically advanced and broadened the range of CT applications from the diagnostic purpose to the navigation system in orthopedic surgery. These CT data can be used for HSA without additional radiation exposure to patients. While some studies have suggested the superior precision in HSA with QCT compared to DXA-HSA (Ramamurthi et al. 2012), it remains to be seen whether this is true for the analysis by utilizing CT data of general clinical practice. Thus, one of the major research questions in this study is to compare the performance of HSA between DXA and CT that has been scanned for the purpose other than HSA.

In this paper, *in vivo* comparison of DXA-HSA to HSA based on QCT (CT-HSA) is reported in a population of postmenopausal Japanese women. Additionally, the biomechanical significance of these two kinds of HSA were evaluated by comparing results to the CT-based finite-element method (CT/FEM), which is considered one of the gold standards for noninvasive assessment of bone strength (Bessho et al. 2007; Keyak et al. 1998; Keller 1994).

## Materials and methods

### Subjects

The subjects were 184 postmenopausal Japanese women who underwent artificial joint replacement surgery for osteoarthrosis of the hip or knee joint between August 2010 and August 2012 in Kawasaki Medical School Hospital. Because 3D CT has been routinely utilized to assist artificial joint replacement surgery in our hospital, CT from the pelvis to the leg was performed in all subjects. Subjects who had implants in the hip joint, bilateral hip osteoarthrosis, or rheumatoid arthritis were excluded. Subjects with osteoporosis were not excluded irrespective of the use of anti-osteoporotic medications. The study was reviewed and approved by the research ethics committee at Kawasaki Medical School. Written informed consent to participate was obtained from all subjects.

For all subjects, CT examination of the lower extremities was performed at an outpatient clinic. Thereafter DXA was performed between the date of admission and the date of surgery. The hip on the non-operative side was analyzed because painful claudication can affect artifactual HSA errors on the affected side.

### DXA and DXA-HSA

BMD was measured at the lumbar spine (L1–L4), femoral neck, and total hip region with DXA using a QDR Discovery A (Hologic Inc., Bedford, MA). Hip DXA was performed at 20 degrees of internal rotation of the hip. DXA-HSA measurements at the narrow neck (NN) and intertrochanteric (IT) regions were made on the standard posterior-anterior (PA) DXA hip image using APEX 3.0 software (Hologic, Inc.). The ROI was determined as follows: 2 pixels above the edge of acetabulum, 2 pixels lateral to the edge of major trochanter, 2 pixels medial to the edge of acetabulum and 20 pixels below the minor trochanter. Among variables computed by HSA programs the following four were used in the present analysis: bone cross-sectional area (CSA), section modulus (SM), average cortical thickness (CTh), and buckling ratio (BR) (Beck et al. 1990).

### QCT

A multi-detector-row CT scanner (Lightspeed Ultra 16, GE Healthcare, Inc., Waukesha, WI) was used. The subjects were scanned from Jacoby line to the toe covering both hip joints, in the supine position, with a calibration phantom B-MAS 200 (Kyoto-Kagaku Co. Ltd., Kyoto, Japan) containing hydroxyapatite at 0, 50, 100, 150, and 200 mg/cm^3^ placed behind the hip. The QCT technique factors were 120 kV, auto mA (noise index: 14), and 2.5 mm slice thickness. We used CT data scanned for the surgical navigation system. Thus, the CT scanning protocol for the navigation system has been adopted.

### Analysis of CT-HSA

**Figure 1:**
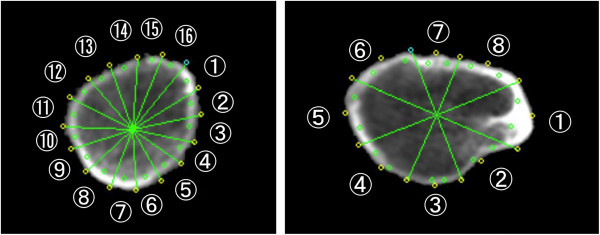
**Schematic representation of sectors in the narrow neck and intertrochanteric regions for the calculation of average cortical thickness (CTh).** Average in cortical thickness in eight or 16 sectors are used for the estimate of CTh in each region.

**Figure 2:**
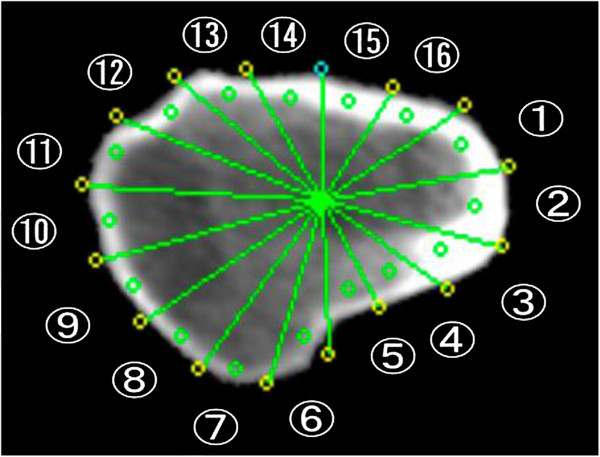
**Schematic representation of 16 sectors in the intertrochanteric region.** Average of buckling ratio (BR) in all sectors is used for the estimate of BR in the intertrochanteric region.

All subjects’ QCT data were processed using QCTPro Software Version 4.1.3 and the QCTPro Bone Investigational Toolkit (BIT Version 2.0, Mindways Software Inc.). Data were evaluated with the CTXA Hip Exam Analysis protocol (Mindways Software Inc.). QCT BIT processing was then performed with a fixed bone threshold for inner cortical separation, which was set to 350 mg/cm^3^ for all of the CT images. The VOI was positioned from three slices above the edge of acetabulum to 10 slices (25 mm) below the minor trochanter. The Narrow-Neck Series was used in the neck region, and Hip Strength Processing was used in the IT region. In the neck region, CSA, CTh, SM (Zmax), and BR were calculated for all 11 slices, and an average value of five consecutive slices at the trochanteric side was used in this study because of the artifact of acetabulum and/or ischium. Since femoral neck length is shorter in Japanese than in Caucasian, in some cases the edge of femoral neck is difficult to separate from acetabulum and/or ischium even with CT. To avoid this possible error, we used five consecutive slices at the trochanteric side that is also recommended in the manual of QCTPro. In the IT region, CSA, CTh, SM (Z2), and BR were calculated. CTh was calculated as an average of thickness in each sector (NN, sectors 1–16; IT, sectors 1–8) (Figure 1). Since the output of QCTPro does not calculate BR in the IT region, it was calculated using Dump (Mindways Software Inc.) as follows: Sector_N_BR = Sector_N_AvgCorticalToCM (average cortical to center mass) / Sector_N_ACT (N: sector 1–16) (Figure 2). Each BR in sectors 1 to 16 was averaged to calculate BR in the IT region. Since QCTPro calculates several different indices of CSA, the index equivalent to DXA-HSA was chosen in the present study.

### CT/FEM

QCT data for all subjects were processed using Mechanical Finder Version 6.0 (Research Center of Computational Mechanics, Inc., Tokyo, Japan) as described previously (Bessho et al. 2007). Young's modulus and yield stress of each tetrahedral element were calculated using the equations proposed by Keyak et al. (Keyak et al. 1998) and Keller (Keller 1994). Poisson's ratio for each element was set as 0.4. Nonlinear FE analysis was performed using the Newton–Raphson method. The loading direction was defined as the angle γ with reference to the long axis of the femur in the frontal plane and δ with reference to the femoral neck axis in the horizontal plane. Then, γ=160° and δ=0° were assigned as stance configuration (SC), and γ=60° and δ=15° were assigned as fall configuration (FC). Fracture load (FL) was defined as the load when ≥1 shell element failed (Bauer et al. 2006).

### Statistical analysis

Data for continuous variables are presented as mean ± standard deviation (SD). Pearson's product–moment correlation coefficient was used for bivariate correlations between continuous variables, DXA-HSA, CT-HSA, and FL. Furthermore, forward stepwise multiple linear regression analysis was used to evaluate to what extent the variation in different FL measures could be explained by DXA-HSA geometry, CT-HSA geometry, age, body weight, height and total hip BMD. Variables not following a normal distribution were logarithmically transformed. Results from the stepwise regression models are presented as standardized regression coefficient, 95%, CI and R^2^. A p-value <0.05 was considered statistically significant. Statistical analysis was performed using SPSS for Windows version 19.0 (SPSS Inc., Chicago, IL).

## Results

Characteristics of the study subjects are outlined in Tables 1 and 2. Thirty-one subjects had a past history of osteoporotic fractures, and 26 subjects reported taking an oral bisphosphonate or selective estrogen receptor modulator.

**Table 1:** **Subject characteristics**

	Mean	SD	Range
Age (years)	70.5	8.7	51 - 88
Weight (kg)	58.8	10.0	38.3 - 94.4
Height (cm)	150.9	5.9	136.4 - 170.0
BMI (kg/m^2^)	25.8	4.2	17.8 - 43.2
FL_SC_ (N)	4400	988.9	2500 - 8050
FL_FC_ (N)	1477	352.2	750 - 2450

*BMI:* body mass index, *FL:* fracture load, *SC:* stance configuration, *FC:* fall configuration.

**Table 2:** **BMD in each skeletal site**

	BMD (g/cm^2^)	%YAM (%)	T-score
Lumbar spine	0.881 ± 0.177	87.1 ± 17.5	−1.1 ± 1.5
Femoral neck	0.592 ± 0.100	74.9 ± 12.7	−2.2 ± 1.1
Total hip	0.742 ± 0.116	84.8 ± 13.2	−1.3 ± 1.2

*BMD:* bone mineral density, *YAM:* young adult mean.

**Figure 3:**
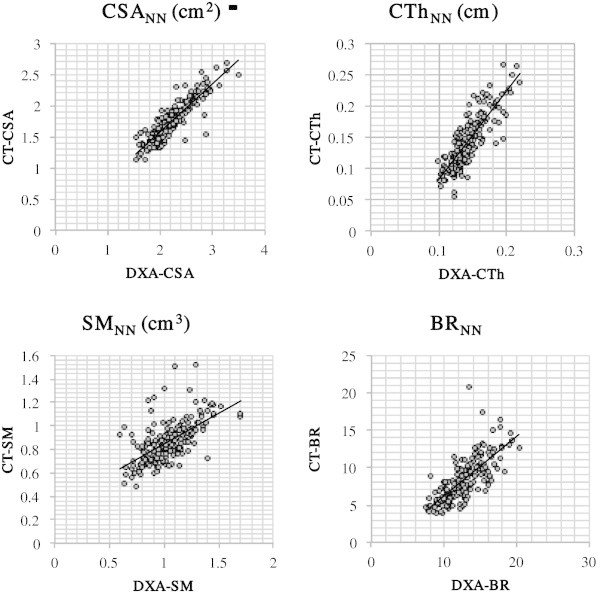
**Scatter plots showing the relationship between DXA-HSA and CT-HSA in the narrow neck region.** CSA: bone cross-sectional area, SM: section modulus, CTh: average cortical thickness, BR: buckling ratio, NN: narrow neck region.

**Figure 4:**
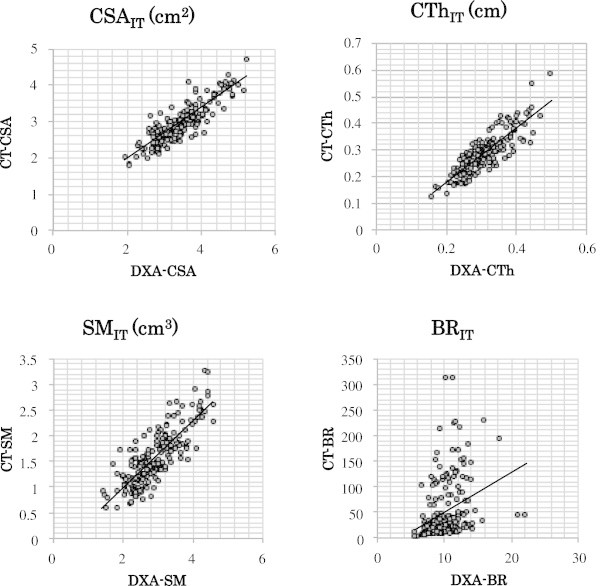
**Scatter plots showing the relationship between DXA-HSA and CT-HSA in the intertrochanteric region.** CSA: bone cross-sectional area, SM: section modulus, CTh: average cortical thickness, BR: buckling ratio, IT intertrochanteric region.

DXA-HSA indices were significantly correlated (p<0.01) with CT-HSA both in the NN and IT regions (Table 3, Figures 3, and 4). Correlation coefficients were high (>0.8) especially in CSA_NN_, CTh_NN_, CSA_IT_, CTh_IT_, and SM_IT_.

**Table 3:** **Correlations between DXA-HSA and CT-HSA**

Region	CSA	CTh	SM	BR
NN	0.90*	0.85*	0.60*	0.74*
IT	0.86*	0.85*	0.82*	0.32*

Values are Pearson's correlation coefficient.

*CSA:* bone cross-sectional area, *CTh:* average cortical thickness, *SM:* section modulus, *BR:* buckling ratio, *NN:* narrow neck region, *IT* intertrochanteric region.

*p<0.001.

DXA-HSA indices as well as CT-HSA indices were significantly correlated (p<0.01) with FLs of SC and FC both in the NN and IT regions (Table 4). In the NN region the correlation between FL and HSA indices was similar for FL_SC_ and FL_FC_, whereas in the IT region the correlation was generally higher for FL_FC_ than for FL_SC_. Overall, the correlation between FL and HSA was similar or even higher in DXA-HSA compared to CT-HSA. As for BR, the correlation was significantly higher in DXA-HSA compared to CT-HSA.

**Table 4:** **Correlation between FL and HSA indices**

	CSA	CTh	SM	BR
DXA-HSA in NN				
FL_SC_	0.70*	0.70*	0.49*	−0.52*
FL_FC_	0.68*	0.66*	0.60*	−0.52*
DXA-HSA in IT				
FL_SC_	0.66*	0.64*	0.60*	−0.54*^#^
FL_FC_	0.78*	0.73*	0.71*	−0.65*^#^
CT-HSA in NN				
FL_SC_	0.67*	0.61*	0.38*	−0.47*
FL_FC_	0.69*	0.56*	0.52*	−0.45*
CT-HSA in IT				
FL_SC_	0.62*	0.64*	0.60*	−0.24*^#^
FL_FC_	0.77*	0.71*	0.68*	−0.29*^#^

Values are Pearson's correlation coefficient.

*CSA:* bone cross-sectional area, *SM:* section modulus, *CTh:* average cortical thickness, *BR:* buckling ratio, *NN:* narrow neck region, *IT* intertrochanteric region, *FL:* fracture load, *SC:* stance configuration, *FC:* fall configuration.

*p<0.001.

^#^Significantly different between HSA-DXA and CT-DXA (p<0.001).

Total hip BMD was significantly correlated with FL and the correlation coefficient was 0.66 and 0.77 in stance and fall configuration, respectively (p<0.01).

Tables 5 and 6 show the results of multiple linear regression analysis of FL with HSA indices in several models. In all models, CSA or SM was an independent predictor of FL, and R^2^ was similar (and even higher) in HSA-DXA for FL_SC_.

**Table 5:** **Multiple linear stepwise regression analysis with FL**_**SC**_**as the dependent variable**

	Regression coefficient		Model	
	β	p value	R^2^	p
Model 1			0.59	<0.001
CSA_NN_	1.084	<0.001		
SM_NN_	−0.621	<0.001		
SM_IT_	0.224	<0.01		
Model 2			0.625	<0.001
CSA_NN_	0.999	<0.001		
SM_NN_	−0.573	<0.001		
Age	−0.218	<0.001		
SM_IT_	0.205	<0.01		
Model 3			0.549	<0.001
CSA_NN_	0.704	<0.001		
SM_IT_	0.333	<0.001		
SM_NN_	−0.292	<0.001		
Model 4			0.586	<0.001
CSA_NN_	0.692	<0.001		
SM_IT_	0.239	<0.001		
SM_NN_	−0.317	<0.001		
Age	−0.152	<0.01		
Height	0.116	<0.05

**Table 6:** **Multiple linear stepwise regression analysis with FL**_**FC**_**as the dependent variable**

	Regression coefficient		Model	
	β	p value	R^2^	p
Model 1			0.600	<0.001
CSA_IT_	0.776	<0.001		
Model 2			0.630	<0.001
CSA_IT_	0.812	<0.001		
Age	−0.148	<0.01		
Weight	−0.132	<0.05		
Model 3			0.644	<0.001
CSA_IT_	0.415	<0.001		
CSA_NN_	0.261	<0.001		
SM_IT_	0.222	<0.01		
Model 4			0.678	<0.001
CSA_IT_	0.406	<0.001		
Total hip BMD	0.428	<0.001		
SM_NN_	0.173	<0.001		
Weight	−0.149	<0.01		

β: standardized regression coefficient, R^2^: adjusted R^2^.

Independent variables included in each model are DXA-HSA indices (CSA_NN_, CTh_NN_, SM_NN_, BR_NN_, CSA_IT_, CTh_IT_, SM_IT_, and BR_IT_) in model 1; DXA-HSA indices and total hip BMD, height, weight, and age in model 2; CT-HSA indices (CSA_NN_, CTh_NN_, SM_NN_, BR_NN_, CSA_IT_, CTh_IT_, SM_IT_, and BR_IT_ ) in model 3; CT-HSA indices, total hip BMD, height, weight, and age in model 4.

*CSA:* bone cross-sectional area, *SM:* section modulus, *CTh:* average cortical thickness, *BR:* buckling ratio, *NN:* narrow neck region, *IT* intertrochanteric region, *FL:* fracture load, *SC:* stance configuration, *FC:* fall configuration.

## Discussion

Although BMD (in grams per square centimeter as measured by DXA) is the current gold standard for clinical assessment of bone fragility (Cummings et al. 2002; Marshall et al. 1996), it is well known that BMD itself is inadequate for accurate estimation of bone strength (Cody et al. 1999; Esses et al. 1989; Beck et al. 1990). It is necessary to evaluate strength considering not only BMD but also bone quality. Among several factors contributing to bone quality, the geometry of the proximal femur can be assessed using an HSA program in routine hip DXA measurement. This technique is expected to have a supplemental role in assessment of bone strength.

It has been reported that Asians generally have a lower BMD than Caucasians, but hip fracture rates are lower in Asians (Villa et al. 2001; Xu et al. 1996; Lau et al. 2001; Ross et al. 1991). The size and geometry of proximal femur could partially account for this difference across ethnicities. Indeed, a comparative study between Japanese and Caucasian American women showed that Japanese women had shorter femoral neck and smaller femoral neck angle compared to Caucasian women, which was associated with a lower risk of structural failure (Nakamura et al. 1994). This characteristic of a shorter femoral neck in Japanese women can sometimes lead to inaccurate hip DXA measurement. Before the present study, there had been no report on the accuracy of HSA assessment in Japanese subjects.

First, HSA indices were compared based on DXA, with the equivalent indices calculated on QCT. Overall, DXA-HSA was highly correlated with CT-HSA. The correlation had an r>0.8, except for SM in the NN region and for BR in the NN and IT regions. This is consistent with the recent report by Ramamurthi et al. (Ramamurthi et al. 2012), in which DXA-HSA was compared with measurements obtained by high-resolution QCT. They showed that DXA-HSA correlates strongly with CT-HSA both in the NN and IT regions (r=0.89-95). Although the correlation in the present study was slightly lower than that reported by Ramamurthi et al., this difference could be attributed to the difference in the registration technique. They used a sophisticated method to ensure that really the same regions were analyzed between CT and DXA. Another possible cause of the difference would be the difference in slice thickness in CT. In the present study, CT data with a 2.5 mm slice thickness was used, parameters which are routinely adopted in preoperative examination of joint replacement surgery in our institute. On the other hand, Ramamurthi et al. used a 1 mm slice thickness. Because even high-resolution QCT cannot accurately measure cortical thickness below 1.0-1.5 mm (Prevrhal et al. 1999), CTh estimated with CT in the present study would have been affected by partial volume averaging, especially in subjects with thin cortices.

Compared to the overall high correlation of HSA between DXA and CT, the correlation of BR_IT_ was significantly lower than the other parameters. Since the mean value of BR was calculated in the present study by averaging the BR of each sector, sectors with a thin cortex could be largely affected by the partial volume effect and result in an error in estimating cortical thickness. Error in cortical thickness is augmented by calculating BR.

When comparing DXA-HSA with CT-HSA, the correlation of HSA with FL estimated by FEM based on QCT (which is one of the current gold standards of *in vivo* assessment of bone strength) was also evaluated. The correlation coefficients between total hip BMD and FL were 0.66 and 0.77 in stance and fall configuration, respectively and were equivalent to the results by Danielson et al. (Danielson et al. 2013) and Orwoll et al. (Orwoll et al. 2009). Both DXA-HSA and CT-HSA were significantly correlated with FL, and the correlation was similar or even higher in DXA-HSA compared to CT-HSA. However, the correlation itself was not very high and did not suggest equivalence. This is consistent with recent reports by Danielson et al. (Danielson et al. 2013), who found that femoral neck geometry computed by HSA from DXA data corresponds well to that from QCT for analysis of load stress in a large cohort of postmenopausal women. They also showed that proximal femur breaking strength estimated from 2D DXA data was not as well correlated with femur breaking strength derived by 3D FEM using QCT data (Danielson et al. 2013). The localized measurement in the proximal femur in the neck or trochanter may not be representative for the total proximal femur.

Several groups have recently published prospective studies assessing the efficacy of HSA to predict hip fractures. Kaptoge et al. analyzed DXA-HSA data from 7,474 women in the prospective population-based Study of Osteoporotic Fractures (SOF) (Kaptoge et al. 2008). In the SOF, 635 women suffered incident hip fractures over 13 years of follow-up. They concluded that for hip fracture prediction using NN region parameters, CTh, areal BMD, and BR were equivalent, but SM performed less well. LaCroix et al. studied 10,290 postmenopausal women from the Women's Health Initiative (LaCroix et al. 2010). They concluded that two hip geometry parameters, outer diameter and BR in the IT region, can predict incident hip fracture after accounting for clinical risk factors and BMD (Ross et al. 1991). In the present study, significant predictors of FL_SC_ in DXA-HSA were CSA_NN_, SM_NN_, SM_IT_, and age; and significant predictors of FL_SC_ in CT-HSA were CSA_NN_, SM_NN_, SM_IT_, age, and height. Significant predictors of FL_FC_ in DXA-HSA were CSA_IT_, age, and weight and significant predictors of FL_FC_ in CT-HSA were CSA_IT_, CSA_NN_, SM_IT_, SM_NN_, total hip BMD, and weight. Thus the results in this study do not correspond well with those of the prospective studies. Although it is difficult to compare many variables that are closely correlated with each other in multiple regression analysis, the discrepancy might suggest the biomechanical assumption does not completely describe our FEM model.

This study had a number of limitations. First, all subjects were postmenopausal women before artificial joint replacement surgery for osteoarthrosis of the hip or knee joint. Because CT has been adopted as a routine preoperative examination for artificial joint replacement surgery in our institute, this method of subject recruitment solved the problem of additional radiation exposure. On the other hand, the subjects may not represent the general postmenopausal Japanese population. It has been reported that subjects with osteoarthrosis may have increased BMD (Dequeker et al. 2003). Alternatively, osteoarthrosis may also be related to increased bone resorption, which results in decreased bone mass (Henrotin et al. 2009) and increased fracture risk. In this study, lumbar and total hip BMDs were higher, whereas femoral neck BMD was close to previous reports (Orimo et al. 2001; Orimo et al. 1998). Second, the methods used to calculate HSA indices are inherently different and limit strict comparison. Finally, QCT data scanned with a slice thickness of 2.5 mm was used, potentially negatively affecting the precision of HSA measurement. However, since reduced slice thickness is associated with a corresponding increase in radiation exposure, reduction of slice thickness is often limited in general clinical practice. The present data suggest that the CT-HSA using the scan condition such as 2.5 mm slice thickness would provide little information over DXA-HSA and the DXA-HSA could be sufficiently accurate compared to such CT methods.

## Conclusion

In conclusion, there was high correlation between DXA-HSA and CT-HSA for CSA, CTh, and SM in postmenopausal Japanese women. Moreover, the correlation of HSA with FL was similar between DXA-HSA and CT-HSA (and was even slightly higher in DXA-HSA). These results suggest that the geometry of proximal femoral cross sections is reasonably well characterized by DXA.
